# *De novo* sequencing of sunflower genome for SNP discovery using RAD (Restriction site Associated DNA) approach

**DOI:** 10.1186/1471-2164-14-556

**Published:** 2013-08-15

**Authors:** Venkatramana Pegadaraju, Rick Nipper, Brent Hulke, Lili Qi, Quentin Schultz

**Affiliations:** 1 BioDiagnostics, Inc, 507 Highland Drive, River Falls, WI 54022, USA; 2 Floragenex, Inc. 44 West Broadway, Eugene, OR 97401, USA; 3 USDA-Agricultural Research Service, Northern Crop Science Laboratory, 1307 18th St. N., Fargo, ND 58102-2765, USA

**Keywords:** Single nucleotide polymorphism (SNP), Restriction site associated DNA sequencing (RAD-Seq)

## Abstract

**Background:**

Application of Single Nucleotide Polymorphism (SNP) marker technology as a tool in sunflower breeding programs offers enormous potential to improve sunflower genetics, and facilitate faster release of sunflower hybrids to the market place. Through a National Sunflower Association (NSA) funded initiative, we report on the process of SNP discovery through reductive genome sequencing and local assembly of six diverse sunflower inbred lines that represent oil as well as confection types.

**Results:**

A combination of Restriction site Associated DNA Sequencing (RAD-Seq) protocols and Illumina paired-end sequencing chemistry generated high quality 89.4 M paired end reads from the six lines which represent 5.3 GB of the sequencing data. Raw reads from the sunflower line, RHA 464 were assembled *de novo* to serve as a framework reference genome. About 15.2 Mb of sunflower genome distributed over 42,267 contigs were obtained upon assembly of RHA 464 sequencing data, the contig lengths ranged from 200 to 950 bp with an N_50_ length of 393 bp. SNP calling was performed by aligning sequencing data from the six sunflower lines to the assembled reference RHA 464. On average, 1 SNP was located every 143 bp of the sunflower genome sequence. Based on several filtering criteria, a final set of 16,467 putative sequence variants with characteristics favorable for Illumina Infinium Genotyping Technology (IGT) were mined from the sequence data generated across six diverse sunflower lines.

**Conclusion:**

Here we report the molecular and computational methodology involved in SNP development for a complex genome like sunflower lacking reference assembly, offering an attractive tool for molecular breeding purposes in sunflower.

## Background

Domestic sunflower (*Helianthus annuus L., 2n* = *2x* = 34, haploid genome size ~3.5 Gbp) is native to North America [1] and widely cultivated as oilseed and confection crop types. Besides being economically important, sunflower also serves as model in ecological and evolutionary studies [2-4]. A major focus in both public and private sunflower breeding programs has been to develop sunflower hybrid varieties with improved yield, oil content and resistance to a wide range of diseases. Breeding new hybrids by conventional practices mostly is slow and uncertain; however, application of molecular markers can improve efficiency of plant selection, saving time and providing accuracy in a breeding program [5-8]. A wide range of molecular markers such as RFLP, AFLP, SSR and TRAP developed in sunflower have successfully enabled construction of high density genetic maps [9-15] and led to identification of molecular markers linked to disease resistance genes [16-19]. However, in general, practical usage of these markers for routine breeding purposes is limited due to high assay cost, low reproducibility, and lack of QTL validation studies [20].

In recent years, SNP markers have gained popularity in crop breeding programs due to their low cost, high throughput efficiency, and abundance. Particularly in association mapping studies, SNPs are the preferred marker type since they involves scanning whole genomes with extremely high marker densities to identify closely linked markers to causal polymorphisms [21,22]. It is estimated that due to low linkage disequilibrium and high haplotype diversity, SNPs in the order of several thousand would be needed to successfully conduct genome wide association analysis in sunflower [23].

Large-scale discovery of genome-wide distributed SNPs can be effectively conducted with the aid of massively parallel, next-generation sequencing (NGS) technologies [24]. Several studies that involve whole genome sequencing (WGS) efforts have led to the successful discovery of SNPs in Arabidopsis [25], humans [26], and Medicago [27]. NGS technologies have also been extended for SNP discovery in large and complex genomes that lack an assembled reference genome [28,29]. A common approach in these situations is the use of a complexity reduction strategy that is designed to selectively interrogate a small percentage of the target genome [30,31]. By restricting sequencing on a smaller fraction of the genome, overall sequencing costs are reduced compared to WGS strategies, while still identifying a large amount of genetic variation. For instance, by using an RNA sequencing approach (RNA-Seq) on tissues from two diverse maize inbred lines, more than 4900 SNPs associated with 2,400 genes were identified and validated [32]. Similarly, previous work in sunflower produced nearly 10,000 SNPs with RNA-Seq [29].

In addition, a cadre of methods have been developed that involve the usage of restriction enzymes on genomic DNA for complexity reduction. These strategies can be at the nucleotide level and are viewed as simple and highly efficient methods in plant and animal genome sequencing studies. One such method, CRoPS (complexity reduction of polymorphic sequences) can overcome the problems associated with highly duplicated regions in complex genomes that hamper the process of SNP identification [33]. Restriction site-associated DNA sequencing (RAD-Seq) is an emerging method for SNP detection in genomes and is based on identifying polymorphic variants adjacent to restriction enzyme digestion sites [34,35]. Application of RAD-Seq for identifying genetic variants has been demonstrated in a variety of species with and without an available reference genome [36,37]. More recently paired-end RAD-Seq (RAD-PE) has been used in a variety of efforts for both genome assembly and SNP marker development [38,39].

Furthermore, RAD-Seq approach has also been exploited in wide range of other studies such as association mapping [40], population genetics inferences [41-44], genetic mapping [35,45,46] and in estimation of allele frequencies [47]. RAD-Seq differs from RNA-Seq in that non-transcribed loci are also sequenced, thus affording us an opportunity to broaden the known SNPs in sunflower to include those outside of transcribed regions. Here we demonstrate the use of paired-end RAD sequencing to enable efficient, cost-effective, high throughput marker development in *H. annuus*, a major oil crop without an assembled genome sequence. Results on the use of this sequence resource for detection of sequence variation and design of SNP marker panels for Illumina Infinium Genotyping Technology (IIGT) are also discussed.

## Results and discussion

### Paired-end RAD-Seq and *de novo* assembly

**Figure 1:**
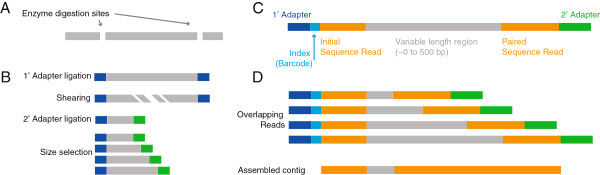
**Paired–end RAD Sequencing Overview. A**. Genomic DNA is digested with a restriction endonuclease. **B**. After ligation with a primary adapter, the fragments are sheared, then ligated with a secondary adapter. **C**. A composite mixture of variable length fragments is recovered from each restriction enzyme digestion site. These fragments are size selected, amplified and sequenced on a next-generation DNA sequencing platform using paired end chemistry. **D**. Development of the genomic assembly around each digestion site is then completed bioinformatically.

Unlike randomized short-insert NGS sequencing methods, RAD genome fragments share a unique architecture: a sequence anchored by the restriction enzyme cleavage site and a variable sequence end generated from a shearing step during library construction (Figure 1). When RAD is coupled with paired-end sequencing approaches now available on NGS platforms, the opposite ends of the RAD fragment are linked in *cis* and the fragment can then be interrogated. Mate-pairs with identical single read sequences can then be readily assembled into contigs spanning hundreds of base pairs (Figure 1C).

To promote SNP identification in low-copy, gene-rich regions of the 3.5 Gbp sunflower genome, a species expected to contain upwards of 80% retrotransposon content [48], the 5-methylcytosine (5mC) sensitive type II nuclease *PstI* (5’CTGCA/G’3) was selected for RAD-Seq in each of the six lines. Numerous studies have documented retroelement-dense regions of plant genome are often subjected to cytosine methylation of CpG, CpNpG and CpNpN nucleotides [49-51]. Restriction enzymes such as *PstI*, which do not cleave 5mC-modified DNA, have been shown to specifically sample the hypomethylated genomic fraction of plant genomes [52].

**Figure 2:**
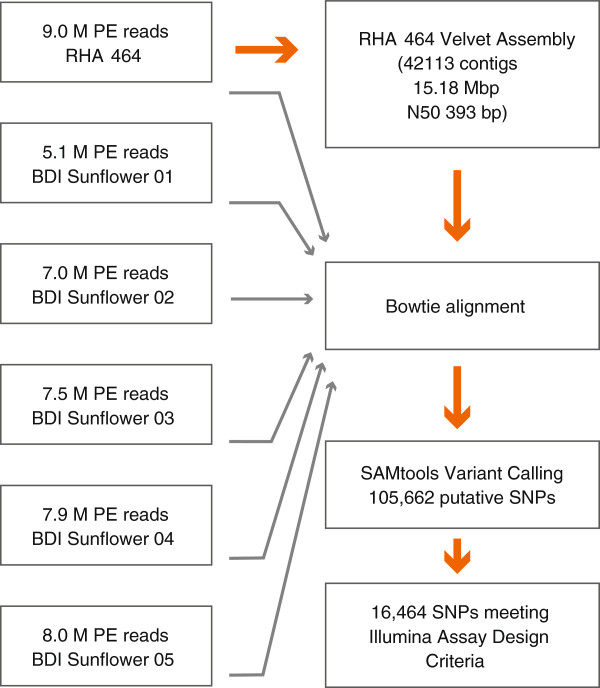
***Helianthus annuus *Sequencing &Analysis Pipeline.** The *de novo* assembly was developed using paired end sequence reads from RHA 464. Bowtie alignments of paired end data from the Helianthus population were used to identify putative SNPs using the SAMtools software suite. A panel of 16,464 variants was ultimately selected for Illumina Infinium Genotyping Design.

**Figure 3:**
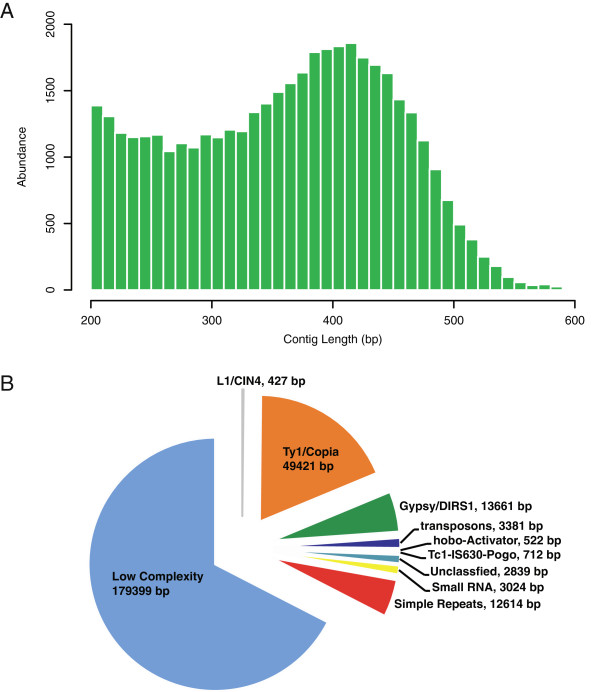
**RAD-Seq Assembly Results and Repeat Element Contribution. A**. The length distribution of RAD-Seq contigs is plotted as a histogram. **B**. The contribution of known repetitive elements in the *H. annuus* RAD sequence assemblies is shown. Results were obtained through RepeatMasker analysis using the Repbase Arabidopsis database.

Sequencing results for each of the six lines are detailed in Additional file 1. A total of 44.7 M reads (89.4 M paired-end) were obtained from all six lines, representing ~5.3 Gb of sequence data. An overall workflow of sequence analysis performed in this study is encapsulated in Figure 2. To construct a skeleton sunflower reference, reads from line RHA 464 [53] were first coalesced into contigs using the Velvet assembler [54]. Initial *de novo* assembly produced ~15.2 Mb of sunflower genome sequence distributed over 42,267 individual contigs. This quantity of assembled sequence data is approximately half of the sequence content developed from a contemporary whole transcriptome sequencing project [55]. Contig lengths for the RHA assembly ranged between 200 and 920 bp with an N_50_ length of 393 bp for all RHA 464 assemblies (Figure 3A and Table 1). The contig length distribution is in line with the fragment size range selected during RAD-Seq library preparation. After initial assembly, contigs were aligned against a custom sequence database to remove sequences with significant plastid homology with 42,113 contigs spanning 15.18 Mbp of the sunflower genome remaining. Contigs passing these filters were then evaluated for the presence of repetitive elements using the RepeatMasker web server with the Arabidopsis Repbase library. The percentage of the RAD-Seq RHA 464 assembly classified as repetitive by RepeatMasker was 1.75%. This is consistent with a genome assembly principally from low-copy regions, as the 3.5 gigabasepair sunflower genome is expected to contain over 80% repetitive nucleotide content. The major classes of repetitive DNA elements that were identified belonged to low complexity sequences and Ty/Copia long-terminal repeat (LTR) retroelement families (Figure 3B and Additional file 2). The GC dinucleotide content for RHA 464 sunflower assembles was 36.2% (Table 1), which is consistent with results from paired-end RAD-Seq studies in other plant genomes [56].

**Table 1:** Paired-end RAD-Seq assembly statistics

**Feature**	**Value**
Number of RHA 464 contigs assembled	42267
Contigs removed due to plastid homology	154
Number of contigs retained	42113
Total assembly length (bp)	15181868
Minimum contig length (bp)	200
Maximum contig length (bp)	920
GC%	36.1
N50 Contig Length (bp)	393
N90 Contig Length (bp)	254

Comparison of RAD-Seq assemblies from RHA 464 to preexisting sunflower unigenes at the Dana Farber Cancer Institute (DFCI) confirmed high sequence identity between RAD assemblies and known sunflower genomic sequences. A representative alignment is shown in Additional file 3 illustrating the match of a single paired-end RAD-Seq contig with tentative consensus EST TC57527 from the *H. annuus* DFCI EST database. The high sequence coverage inherent in paired-end RAD-Seq minimizes sequencing and assembly errors, as each nucleotide in the contig is derived from the consensus of many overlapping Illumina sequence reads. Experimental paired-end RAD-Seq studies using the sequenced B73 maize cultivar have placed the single base accuracy for paired-end RAD-Seq contigs at approximately 99.95% (unpublished data). This accuracy in RAD-Seq contig assembly is a key advantage for applications involving genome assembly and downstream marker development.

### SNP discovery

Alignment of sequence data from all six sunflower inbred lines to the RHA 464 reference used the Bowtie/SAMtools [57,58] variant detection pipeline and revealed the presence of 105,662 putative sunflower SNPs (Table 2). The calculated polymorphism rate in this study is approximately one SNP observed per 143 bp of sunflower genomic sequence. This rate of nucleotide variation agrees well with the levels of genetic diversity reported in recent sunflower studies [29,59,60]. Several additional filtering steps were implemented to pare down the initial SNP dataset to a panel of markers suitable for Infinium genotyping. The full set of 105,662 potential variant loci was scored over the sunflower population. Although SNP genotypes could be reliably called for over 79% of loci (≥ 4x sequence coverage), 11,614 SNPs with missing genotype calls in three or more sunflower lines were not considered for further analysis (Table 2). Genotypes for the remaining 94,048 alleles indicated 89.2% of genotype calls were homozygous in the target population, in line with the inbred nature of the selected lines. The large tracts of sequence landscape generated around candidate SNPs foster the conversion of variants identified from RAD-Seq into downstream genotyping platforms such as the Illumina GoldenGate, Infinium and Sequenom iPlex systems. Of the 94,048 candidate SNPs, less than 4% had to be removed due to the absence of insufficient flanking genomic sequence (minimum limit of 50 bp flanking candidate SNP). The vast majority of discarded SNPs were rejected due to the presence of flanking polymorphisms within 50 bp that can interfere with oligo hybridization during genotyping (Table 2). A final set of 16,467 SNPs was identified and considered for Infinium genotyping design.

**Table 2:** ***Helianthus *SNP filtering and statistics**

**Feature**	**Value**
*Helianthus* samples sequenced:	6
Total number of SNP variants identified:	105662
Total possible SNP genotypes in population:	633972
SNP genotypes with high confidence call:	502837 (79.3%)
SNP genotypes with missing or low quality data:	131135 (20.7%)
SNP loci with < 50% genotype data:	11614
SNPs passing initial filters:	94048
Number of fixed genotype calls:	448289 (89.2%)
Number of heterozygous genotype calls:	54548 (10.8%)
SNP Loci with insufficient flanking sequence for IIGT*:	3445
SNP Loci with nearby polymorphism (< 50 bp):	74136
SNP Loci meeting all defined IIGT assay design criteria:	16467

* Illumina Infinium Genotyping Technology.

**Figure 4:**
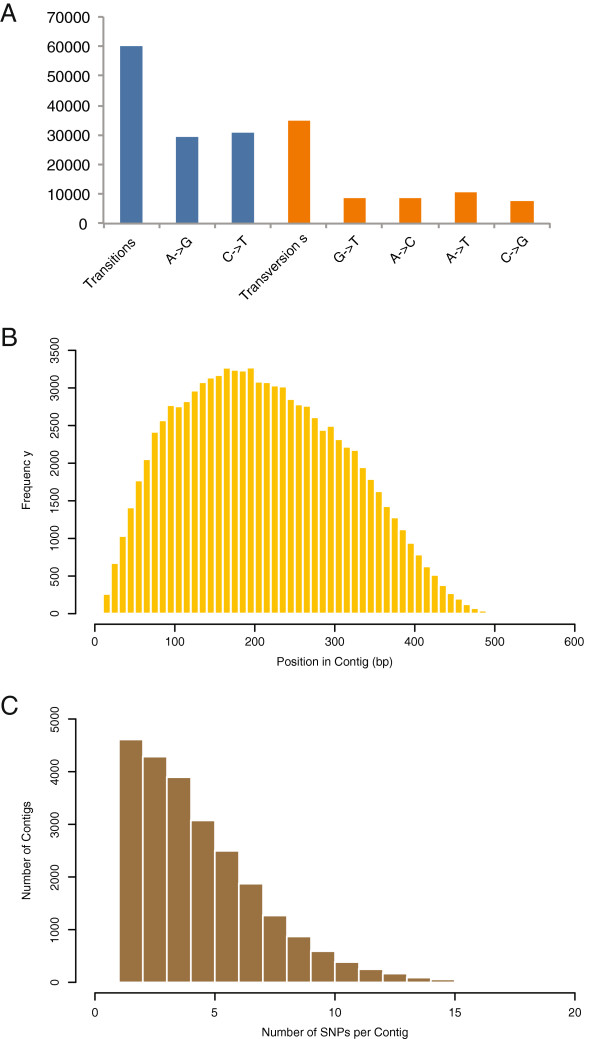
***Helianthus *SNP Discovery. A**. The number and ratio of SNP transitions and transversions observed in the Helianthus population is graphed. **B**. The frequency of SNPs by position in each respective contig is plotted. **C**. The number of sequence variations observed across each RAD-Seq contig is shown.

Analysis of the sequence variation identified in RAD-Seq aligns well with values reported in other plant studies. First, the observed SNP transition/transversion ratio of 1.72 (Figure 4A) in this study is very similar to the distributions reported in other studies of sunflower and eggplant [29,36]. Analysis of the location of variants in assembled RHA 464 contigs indicated no significant bias in allele detection: most SNPs were identified between 100 and 300 bp from the start of each contig (Figure 4B). Finally, approximately 57% of all assembled contigs (23984 out of 42113) contained at least one detectable polymorphism, reflecting the high degree of variation present in sunflower (Figure 4C).

## Conclusions

The application of next-generation DNA sequencing to generate large numbers of genetic markers has revolutionized plant breeding, facilitating both molecular genetic research and marker-assisted selection efforts. We have demonstrated paired-end RAD-Seq is an efficient and cost-effective means of SNP discovery in a species with a complex, highly repetitive genome. From less than a flowcell of Illumina paired-end sequence data we sequenced six diverse sunflower lines, assembled over 40,000 high-quality sequence contigs with an N_50_ contig length of 393 nucleotides, mined more than 100,000 sequence variations from the sunflower genome and identified 16,467 candidate SNPs suitable for downstream genotyping. The RAD-Seq method is appropriate for studies where many thousands of SNPs need to be rapidly identified at a low cost, in a format suitable for high-throughput genotyping.

## Methods

### Plant material and DNA extraction

Sunflower inbred lines (TX16R, CR29, SEEDS2000 B-Line, HA 467, RHA 468, and RHA 464) were grown under laboratory greenhouse conditions for four weeks, all true leaves were harvested and lyophilized prior to DNA extraction. DNA was extracted from 40 mg of each inbred line with the DNeasy 96 Plant Kit (Qiagen) using a modified protocol. Tissue was pulverized with 3 mm beads in a Harbil shaker. Buffer AP1 with DX and RNaseA was added to the tissue, 500 μL per sample, and incubated at 55°C for 60 min. Buffer AP2 was added, 150 μL per well, and incubated at −20°C for 15 min. AP3/E was combined with supernatant, 600 μL and 400 μL respectively, and then added to the binding plates. The rest of the extraction was carried out according to kit instructions. DNA was eluted in a final volume of 50 μL.

DNA was quantified using the PicoGreen kit (Molecular Probes) according to the kit instructions. A standard curve was made using quantified λ DNA from 100 to 0 ng/μL. A 1/200 dilution of Picogreen reagent in 1x TE (provided in kit) was mixed with 2 μL of isolated DNA, briefly vortexed, and incubated in the dark for 5 min. Assays were performed in black 96-well Fluotrac plates and fluorescence was measured with a Spectramax Gemini XPS (Molecular Devices) using 485 nm excitation and 538 nm emission.

### RAD library preparation protocols

Genomic DNA from six selected sunflower inbred lines (TX16R, CR29, SEEDS2000 B-Line, HA 467, RHA 468, and RHA 464) was digested with the restriction endonuclease *PstI* and processed into RAD libraries similar to the method of Baird, et al., 2008. Briefly, ~300 ng of genomic DNA was digested for 60 min at 37°C in a 50 μL reaction with 20 units (U) of *PstI* (New England Biolabs [NEB]). After digestion, samples were heat-inactivated for 20 min at 65°C followed by addition of 2.0 μL of 100 nM P1 Adapter(s), a modified Solexa© adapter (Illumina, Inc.). *PstI* P1 adapters each contained a unique multiplex sequence index (barcode) which is read during the first four nucleotides of the Illumina sequence read. 100 nM P1adaptors were added to each sample along with 1 μL of 10 mM rATP (Promega), 1 μL 10× NEB Buffer 4, 1.0 μL (1000 U) T4 DNA Ligase (high concentration, Enzymatics, Inc), and 5 μL H_2_O which was then incubated at room temperature (RT) for 20 min. Samples were again heat-inactivated for 20 min at 65°C, pooled and randomly sheared with a Bioruptor (Diagenode) to an average size of 500 bp. Samples were then run out on a 1.5% agarose (Sigma), 0.5X TBE gel, and DNA 300 bp to 800 bp was isolated using a MinElute Gel Extraction Kit (Qiagen). End blunting enzymes (Enzymatics, Inc) were then used to polish the ends of the DNA. Samples were then purified using a MinElute column (Qiagen) and 15 U of Klenow exo^−^ (Enzymatics) was used to add adenine (Fermentas) overhangs on the 3′ end of the DNA at 37°C. After subsequent purification, 1 μL of 10 μM P2 adapter, a divergent modified Solexa© adapter (Illumina, Inc.), was ligated to the obtained DNA fragments at 18°C. Samples were again purified and eluted in 50 μL. The eluate was quantified using a Qubit fluorimeter and 20 ng of this product was used in a PCR amplification with 20 μL Phusion Master Mix (NEB), 5 μL of 10 μM modified Solexa© Amplification primer mix (Illumina, Inc.) and up to 100 μL H_2_O. Phusion PCR settings followed product guidelines for a total of 18 cycles. Again, samples were gel purified, excising DNA from the 300 to 700 bp size range, and diluted to 1 nM.

### Illumina sequencing

A set of RAD libraries generated from lines TX16R, CR29, SEEDS2000 B-Line, HA 467, RHA 468, and RHA 464 was run on an Illumina Genome Analyzer IIx at the University of Oregon High Throughput Sequencing Facility in Eugene, Oregon. Illumina protocols were followed for an asymmetric length paired end sequencing run, with an initial 40 bp read and second 80 bp read.

### Bioinformatics – Sequence processing, paired-end RAD-Seq assembly and SNP detection

A combination of open source and proprietary bioinformatics tools was used for processing and sequence analysis. A list of open source programs, versions, and commands used in sequence analysis can be found in a supplemental file (Additional file 4). Initially, raw sequence data produced on two GAIIx sequence lanes were sorted by the appropriate multiplex index (MID) or “barcode” assigned to each sunflower line during RAD-Seq library construction. During de-multiplexing, indexes were trimmed from reads and the remaining sequence segregated to individual sample files. Reads from RHA 464 were then processed to extract low quality sequences. Any sequence with an average phred-scaled quality score below 20 (Q20) over the last 5 base pairs of the read was discarded. Remaining reads were then collapsed into RAD sequence clusters sharing 100% sequence identity across the single end Illumina read. To maximize efficient assembly of sequences we imposed a minimum of 50x and maximum 750x sequence coverage at any RAD sequence cluster. These thresholds were selected for this effort, because single loci with coverage under 50x would be expected to suffer from low sequence coverage ((80 bp × 50)/ 400 bp = 10.0x)) resulting in short and fragmented contig assemblies, while loci with greater than 500 identical SE (Single-end) reads may be composed of high-copy contaminant DNA (plastids) or dosage from multiple genomic loci (e.g. retrotransposon derived sequences). The paired end sequences for each RAD locus were extracted from these selected loci and passed to the Velvet sequence assembler (version 1.0.18) for contig assembly [54]. Contigs not reaching a minimum length of 200 bp were excluded from the assembly.

Sequence reads from TX16R, CR29, Seeds 2000 B-line, HA 467, and RHA 468, were aligned to the reference RHA 464 assembly using the short-read aligner Bowtie (version 0.12.5) [57]. Alignment thresholds were specified which allowed up to three base pair mismatches between the 80 bp Illumina read and the reference (>95% identity). Reads not uniquely mapping (e.g. aligning to more than one contig in the RHA 464 reference) were discarded and not considered in the analysis. Bowtie alignments were piped to SAM tools (version 0.1.14) and reformatted into BAM and pileup files for SNP identification [58]. Sequence variants from pileups were then condensed into a variant call format (VCF) file using custom perl scripts. To be considered for genotyping design, a SNP had to have a minimum sequencing coverage of 4x in at least three lines, with at least 50 bp of flanking genomic sequence surrounding the target SNP. Variants with nearby flanking polymorphisms (within 50 bp of the candidate marker) were also excluded from further consideration for Infinium genotyping design.

## Competing interests

This project was conducted in accordance with the NSA SNP Consortium, a public-private partnership between the non-profit National Sunflower Association, public researchers at USDA, and private seed companies. It was formed with the intent of having widespread, economically feasible application of SNP markers for the advancement of sunflower germplasm. NSA encourages public research entities to use the proprietary markers through NSA approved laboratories for research that meets the public research criteria. Such use will not incur any fees for membership or royalties. For-profit researchers may join the consortium.

## Authors’ contributions

VP designed, coordinated and oversaw the sunflower sequencing, data analysis and drafted the manuscript. RN created RAD libraries for sunflower sequencing and conducted the bioinformatics analysis and assisted in manuscript. BH & LQ were involved in planning the experiments, provided the material for sequencing and preparing the manuscript. QS helped in project planning and provided help in preparing the manuscript. All the authors read and approved the final manuscript.

## Supplementary Material

Additional file 1 Illumina Sequencing Results. Click here for file

Additional file 2 ***Helianthus *RAD-Seq RepeatMasker reports.** Click here for file

Additional file 3 Alignment of RAD-Seq contig with Sunflower EST collection at DFCI. Click here for file

Additional file 4 List of open source programs and commands used in analysis. Click here for file
