# Supplementary material for: De novo sequencing of sunflower genome for SNP discovery using RAD (Restriction site Associated DNA) approach
Source: BMC Genomics. 2013 Aug 15;14:556. doi: 10.1186/1471-2164-14-556 (PMC3765701; doi:10.1186/1471-2164-14-556)
Supplement: Additional file 3 — Alignment of RAD-Seq contig with Sunflower EST collection at DFCI. [file 1471-2164-14-556-S3.pdf]

```
Subject: TC57527

Query: 47481_TGCAGTTGTAACTTAAGCATTTCTATCAA_NODE_1_length_482_cov_13.327801

203 ATCATCCTGGATTTTTCGGTAAAGTTGGTATGAGGTACTTCCACAAGCTTCGCAACAAGT 262

  1 ATCATCCTGGATTTTTCGGTAAAGTTGGTATGAGGTACTTCCACAAGCTTCGCAACAAGT 60

263 TCTATTGCCCTATCGTCAACGTCGACAGGCTCTGGTCGCTTGTGCCACAAGACGTGAAGG 322

 61 TCTATTGCCCTATCGTCAACGTCGACAGGCTCTGGTCGCTTGTGCCACAAGACGTGAAGG 120

323 AGAAGTCTACTGCCGATAAGGTTCCAGTCATTGATGTGACTCAGCACGGTTACTTCAAGG 382

121 AGAAGTCTACTGCCGATAAGGTTCCAGTCATTGATGTGACTCAGCACGGTTACTTCAAGG 180

383 TGTTGGGGAAGGGAAACGTGCCTGCTTCGCAGCCGTTTGTTGTTAAGGCGAAGCTTATTT 442

181 TGTTGGGGAAGGGAAACGTGCCTGCTTCGCAGCCGTTTGTTGTTAAGGCGAAGCTTATTT 240

443 CGAAAACTGCTGAGAAGAAGATTAAGGAGGCTGGTGGTGCTGTTTTGCTCACTGCTTAGG 502

241 CGAAAACTGCTGAGAAGAAGATTAAGGAGGCTGGTGGTGCTGTTTTGCTCACTGCTTAGG 300

503 TTTGTTTTTTTGAATTTGGATGATGAGTATTGGTGTAACTGTTAGTTTTATTGTGAGATT 562

301 TTTGTTTTTTTGAATTTGGATGATGAGTATTGGTGTAACTGTTAGTTTTATTGTGAGATT 360

563 ACGTTGTTCTGATGAATTTGAACTCACATTTTATCAAAGTTTTGTTGCAAAATCCTCAAA 622

361 ACGTTGTTCTGATGAATTTGAACTCACATTTTATCAAAGTTTTGTTGCAAAATCCTCAAA 420

623 TTGTGTTCATTTTCTGCTGATTTTTTGGTGTTTTTGGTTTTA 664

421 TTGTGTTCATTTTCTGCTGATTTTTTGGTGTTTTTGGTTTTA 462
```
